# A Reversible Data Hiding Method in Encrypted Images for Controlling Trade-Off between Hiding Capacity and Compression Efficiency

**DOI:** 10.3390/jimaging7120268

**Published:** 2021-12-07

**Authors:** Ryota Motomura, Shoko Imaizumi, Hitoshi Kiya

**Affiliations:** 1 Graduate School of Science and Engineering, Chiba University, 1-33 Yayoicho, Chiba 263-8522, Japan; ryo.0098@chiba-u.jp; 2 Graduate School of Engineering, Chiba University, 1-33 Yayoicho, Chiba 263-8522, Japan; 3 Department of Computer Science, Tokyo Metropolitan University, 6-6 Asahigaoka, Tokyo 191-0065, Japan; kiya@tmu.ac.jp

**Keywords:** reversible data hiding, image encryption, encryption-then-compression system, lossless compression, hiding capacity

## Abstract

In this paper, we propose a new framework for reversible data hiding in encrypted images, where both the hiding capacity and lossless compression efficiency are flexibly controlled. There exist two main purposes; one is to provide highly efficient lossless compression under a required hiding capacity, while the other is to enable us to extract an embedded payload from a decrypted image. The proposed method can decrypt marked encrypted images without data extraction and derive marked images. An original image is arbitrarily divided into two regions. Two different methods for reversible data hiding in encrypted images (RDH-EI) are used in our method, and each one is used for either region. Consequently, one region can be decrypted without data extraction and also losslessly compressed using image coding standards even after the processing. The other region possesses a significantly high hiding rate, around 1 bpp. Experimental results show the effectiveness of the proposed method in terms of hiding capacity and lossless compression efficiency.

## 1. Introduction

Image data hiding, which is a common security technique for digital images, embeds secret information into a cover image imperceptibly and has attracted attention over recent decades in the image security field. In particular, reversible data hiding (RDH) can perfectly retrieve original images when the embedded payload is correctly extracted [1,2,3,4,5,6,7,8,9]. It is effective not only for natural images but also for medical, military, evidential images, and so forth. Numerous RDH techniques have been proposed for plain images. In recent years, RDH in encrypted images (RDH-EI) has been actively studied [10,11,12,13,14,15,16,17,18,19,20,21,22]. A high hiding capacity is one of the common requirements in the field of data hiding techniques. If the hiding capacity is large enough, we can embed not only simple copyright information but also a certificate and other important documents. It is difficult to visually recognize encrypted images; in addition to the above common payload, it is required for RDH-EI to allow us to embed information on image content, e.g., categorical data and annotation data, so as to be aware of what kind of images they are without decryption. On another front, an image user may desire to obtain original images or high-quality images that still contain hidden information. RDH-EI methods effectively satisfy such requirements.

An RDH-EI method proposed by Zhang [10] encrypts a whole image pixel-by-pixel using the exclusive-or operation. Hong et al. [11] further modified Zhang’s method [10]. With these methods, however, the payload cannot be perfectly extracted. Another work [12] has extended Zhang’s method [10] to improve the marked-image quality. Nevertheless, the payload cannot be correctly extracted if the payload amount becomes large. Ma et al.’s method [13] provides perfect reversibility. This method first divides an image into two regions in the spatial domain. The least significant bits (LSBs) in one region are embedded into another region, and the entire image is then encrypted by the exclusive-or operation. Thus, the LSBs in the former region are referred to as the embeddable area. A data hider embeds payload bits into the encrypted embeddable area by LSB substitution. With this method, an original image can be restored after only decryption without data extraction. This is because the original LSBs, which would be replaced with payload bits, have been stored in another region. A hiding capacity of up to 0.5 bpp has been attained.

Many RDH-EI methods have been studied to achieve a high hiding capacity [14,15,16,17,18,19,20]. For example, Puteaux et al. [14] introduced most significant bit (MSB) prediction and replacement instead of using LSBs. This method does not require any complex processes. Dragoi et al. [15] enhanced the security of [14], and Puteaux et al. [17] further improved the capacity of [14]. However, there still exist multiple cases where reversibility is not fully ensured. Hirasawa et al. [18], whose method is referred to as the RDH-MSB method, extended Puteaux et al.’s method [14] to guarantee full reversibility by defining precise conditions. Wu et al.’s method [19] attained a higher hiding capacity of 2.2 bpp on average by utilizing Puteaux et al.’s method [14]. Further, Puteaux et al. [20] proposed a new RDH-EI method with a higher capacity that guarantees full reversibility and high security. The hiding capacity is 2.4 bpp on average through the recurrent use of MSB prediction. These high-capacity RDH-EI methods, however, cannot decrypt marked encrypted images without data extraction. Additionally, it is difficult for the previous RDH-EI methods to compress their marked encrypted images.

The first method to obtain effective compressibility for marked encrypted images is Imaizumi et al.’s method [21]. This method uses an encryption-then-compression (EtC) system [23,24] and thus can losslessly compress marked encrypted images using image coding standards, such as JPEG-LS [25] and JPEG 2000 [26]. Since an image histogram is not transformed before/after the encryption processes, flexible data hiding and extraction in plain and/or encrypted domains are accomplished by using an RDH method based on histogram shift (RDH-HS) [2]. This method, however, has an extremely low hiding capacity, i.e., about 0.1 bpp. Hereafter, we call this method the RDH-EtC method.

In this paper, we propose a new framework for RDH-EI that can control both the hiding capacity and lossless compression efficiency. Our method tackles the main issue of the RDH-EtC method, namely, the low hiding capacity, without compromising its features. We developed this RDH-EtC framework so that marked encrypted images can be stored with the minimum amount of data while maintaining the required hiding capacity. That could lead to cost savings for storage and data transmission. First, an original image is arbitrarily classified into two regions. The RDH-EtC method is used for one region, and the RDH-MSB method is used for the other region. The former region can be decrypted without data extraction and also compressed by using image coding standards. For the latter region, the hiding capacity is significantly high. Thus, we can choose the embedding region depending on information attributes. Note that there is a trade-off between the hiding capacity and compression efficiency. Through our experiments, we confirm the effectiveness of the proposed method in terms of hiding capacity and lossless compression performance using JPEG-LS and JPEG 2000.

## 2. Related Work

As mentioned, the proposed method arbitrarily divides an original image into two regions, with the RDH-EtC method used for one region and the RDH-MSB method used for the other. We explain the RDH-EtC and RDH-MSB methods as follows.

### 2.1. RDH Method for EtC Images

**Figure 1:**
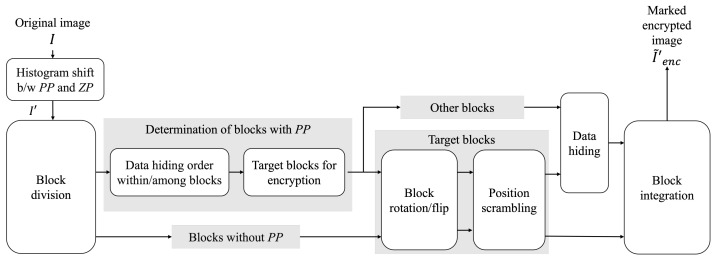
Block diagram of RDH-EtC method [21]. PP and ZP are bins with the highest and lowest frequency bins in the original-image histogram.

In the RDH-EtC method [21], the compression efficiency of marked encrypted images is well considered. Additionally, we can freely embed and extract a payload in plain and/or encrypted domains. This feature indicates that the RDH-EtC method can derive a marked image by decrypting without data extraction. The RDH-EtC method uses two of four processes in the block scrambling-based encryption for EtC systems [23,24]: position scrambling and block rotation/flip. Since these two scrambling processes do not transform image histograms, we can flexibly embed and extract a payload by using the RDH-HS method [2]. Consequently, this method can losslessly compress marked encrypted images by using image coding standards, such as JPEG-LS [25] and JPEG 2000 [26]. Further, it can embed a payload in plain and/or encrypted domains and flexibly extract the payload from either domain. Thus, the data hiding and extraction domain is selectable depending on the user’s request. A marked image can also be derived by decrypting with a payload. However, this method has a significantly low hiding rate of about 0.07 bpp at maximum for grayscale images. We explain the procedures of the RDH-EtC method in accordance with Figure 1.

**Step1-1:**  Perform preprocessing on an original image *I* to prevent overflow and underflow, and an intermediate image $I^{\prime }$ is obtained.
**Step1-2:**  Divide $I^{\prime }$ into multiple blocks with $B_{x}\times B_{y}$ pixels.
**Step1-3:**  Determine the data hiding order within/among the blocks according to the defined conditions.
**Step1-4:**  Extract target blocks for block rotation/flip and position scrambling according to the defined conditions.
**Step1-5:**  Perform block rotation/flip and position scrambling on the target blocks defined in Step 1–4.
**Step1-6:**  Embed a payload into the target blocks in the data hiding order defined in Step 1–5.
**Step1-7:**  Integrate all the blocks, and a marked encrypted image ${\tilde{I}}_{enc}^{\prime }$ is derived.

By switching the encryption and data hiding processes, i.e., Steps 1–5 and 1–6, we can first embed a payload into an original image and then encrypt the intermediate image.

### 2.2. MSB Prediction Based RDH Method

**Figure 2:**
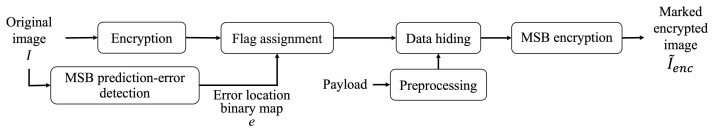
Block diagram of the RDH-MSB method [18].

The RDH-MSB method [18] has attained a high hiding capacity through the introduction of MSB prediction and substitution. Further, the mathematical complexity is relatively low. First, MSB prediction errors are detected from an original image, and the whole image is then encrypted by the exclusive-or operation. In the encrypted image, embeddable and unembeddable pixels are discriminated by assigning flags. A payload is embedded into the embeddable pixels by MSB replacement. LSB replacement is commonly used for data hiding such as used in Ma et al.’s method [11]. However, LSBs among adjacent pixels typically have no correlation. Thus, it is difficult to achieve both reversibility and a high hiding capacity with LSB replacement. In contrast, MSBs among adjacent pixels tend to be highly correlated with each other. This method utilizes the correlation among MSBs and has achieved a high hiding capacity of about 1 bpp. However, it uses pixel-based encryption and thus cannot compress marked encrypted images. Additionally, the payload must be extracted in the encrypted domain, and the decryption process cannot be conducted without data extraction. An outline of the RDH-MSB method is shown in Figure 2. We describe the procedure in detail as follows.

**Step2-1:**  Detect MSB prediction errors in the plain domain using adjacent pixels, and store the errors in an error location binary map *e*.
**Step2-2:**  Encrypt an original image *I* using the exclusive-or operation with a pseudo-random number sequence.
**Step2-3:**  Divide the encrypted image and *e* into blocks with 8×1 pixels and 8 bits, respectively.
**Step2-4:**  In accordance with *e*, if one or more prediction errors are identified in a block, exclude the block from embedding and substitute the MSBs with the values of *e*. In the meantime, the blocks without prediction errors are defined as embeddable blocks.
**Step2-5:**  Assign flags to the first and final blocks in each sequence of embeddable blocks.
**Step2-6:**  In each embeddable block, replace the MSBs of the eight pixels with payload bits.
**Step2-7:**  Encrypt the entire MSBs, and derive a marked encrypted image ${\tilde{I}}_{enc}$.

## 3. Proposed Method

Our proposed framework for RDH-EI controls both the hiding capacity and compression efficiency. First, an original image is arbitrarily classified into two regions: region α and region β. The RDH-EtC method [21] is used for region α. Here, the proposed method replaces the encryption process in [21] with Chuman et al.’s method [27], where the block scrambling-based encryption for EtC systems [23,24] is extended to enhance security. Note that the color conversion in [27] is not performed in our method to ensure full reversibility. In contrast, the RDH-MSB method [18] is used for region β. Accordingly, region α can be compressed after encryption and also be decrypted without data extraction, while the hiding capacity is low. In contrast, region β has a high hiding capacity of around 1 bpp, while the features in α are compromised.

In the proposed method, any arbitrary high-capacity RDH-EI method can be used instead of the RDH-MSB method. For instance, we could further enhance the hiding capacity by adopting Puteaux et al.’s method [20], which has a higher hiding capacity of 2.4 bpp on average. We describe the procedure and effectiveness of the proposed method.

### 3.1. Framework of Proposed Method

The proposed method utilizes the features of the related work and controls both the hiding capacity and lossless compression efficiency. First, it arbitrarily classifies an original image into two regions. In one region, the RDH-MSB method is used to enhance the hiding capacity. In the other region, the RDH-EtC method is used to achieve highly efficient lossless compression. Additionally, the proposed method can decrypt the latter region without data extraction. This means that marked images can be derived with our method. We will precisely compare the proposed method with the related work in Section 4.2.

**Figure 3:**
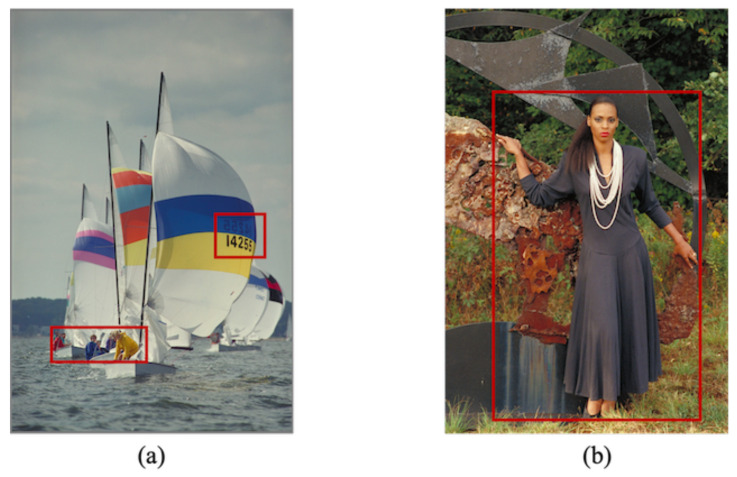
Classification for region α and region β. Region α is areas inside red frames, and region β is other areas. (**a**) kodim09, (**b**) kodim18.

We assume that there are two regions in an image referred to as region α and region β. In Figure 3, for example, the areas marked in red indicate region α.

### 3.2. Procedure of Proposed Method

**Figure 4:**
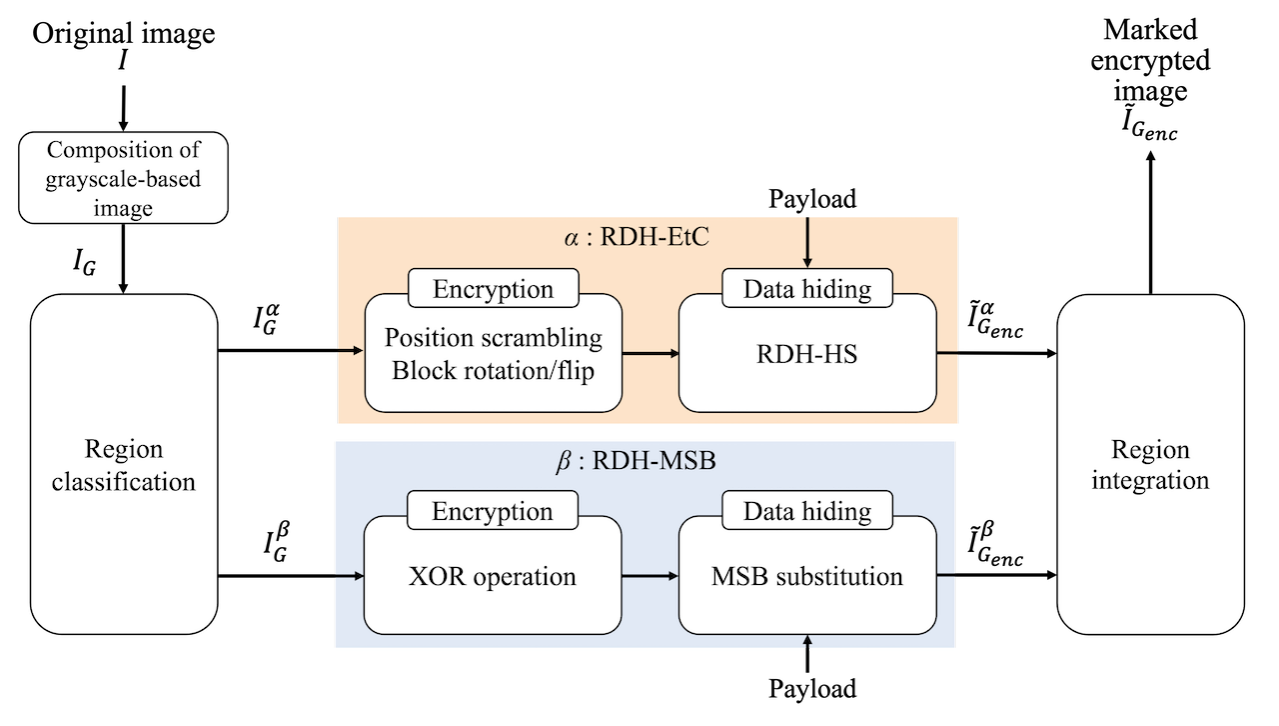
Block diagram of the proposed method.

Figure 4 illustrates the procedure of the proposed method. We explain each step as follows.

1. Combine R, G, and B components of an original image *I*, and a grayscale-based image $I_{G}$ is obtained.
2. Classify $I_{G}$ into region α and region β, and define them as $I_{G}^{\alpha }$ and $I_{G}^{\beta }$, respectively.
3. Perform the RDH-EtC method [21] for $I_{G}^{\alpha }$, and ${\tilde{I}}_{G_{enc}}^{\alpha }$ is derived.
4. Perform the RDH-MSB method [18] for $I_{G}^{\beta }$, and ${\tilde{I}}_{G_{enc}}^{\beta }$ is derived.
5. Integrate ${\tilde{I}}_{G_{enc}}^{\alpha }$ and ${\tilde{I}}_{G_{enc}}^{\beta }$ into the marked encrypted image ${\tilde{I}}_{G_{enc}}$.

In Step 1, the order and direction in which to concatenate the color components are arbitrarily defined. Since region α uses the RDH-EtC method, we can first embed a payload and then encrypt the marked α. Therefore, a user can flexibly embed the payload in plain and/or encrypted domains in α. In Step 2, the classification of regions α and β is defined arbitrarily. Figure 3 is an example of classification, where region α is the areas marked in red. We will describe how the user finds information on the two regions from a marked encrypted image in Section 4.1.

### 3.3. Effectiveness of Proposed Method

The proposed method carefully considers the flaws of the previous pieces of work and makes effective use of their advantages. The hiding capacity, which is seriously low in the RDH-EtC method, is enhanced by introducing the RDH-MSB method. The maximum hiding rate of the RDH-EtC method is 0.07 bpp. In contrast, the hiding capacity is approximately 1 bpp in the RDH-MSB method. When the RDH-MSB method is used for 10% of the spatial domain in a target image, the total hiding capacity for the entire encrypted image is improved to around 0.1 bpp without counting the capacity of the other 90%. It is larger than the maximum hiding capacity of the RDH-EtC method only. Therefore, the proposed method can enhance the hiding capacity as the area where the RDH-MSB method is used increases. Further, our method can use an arbitrary high-capacity RDH-EI method instead of the RDH-MSB method. For instance, Puteaux et al. improved the RDH-MSB method and attained a higher hiding capacity of 2.4 bpp on average [20]. By using this method, we could further enhance the hiding capacity.

In the RDH-MSB method, a marked encrypted image cannot be compressed by image coding standards. Our method improves on this by introducing the RDH-EtC method. The RDH-MSB method uses pixel-based encryption and does not consider the compression performance of marked encrypted images. Conversely, the RDH-EtC method introduces block scrambling-based encryption and thus can losslessly compress marked encrypted images using JPEG-LS [25] and JPEG 2000 [26]. In the proposed method, the region processed by the RDH-EtC method can be greatly compressed, while the region processed by the RDH-MSB method cannot be effectively compressed. Overall, marked encrypted images can be compressed to some degree. The compression performance would be enhanced by assigning the RDH-EtC method to a wider region. Note that there is a trade-off between the hiding capacity and compression efficiency.

**Figure 5:**
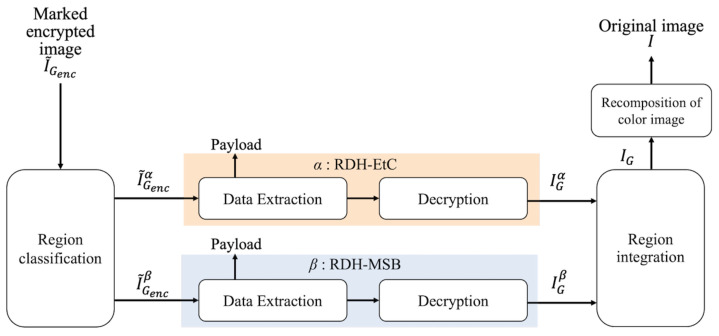
Restoration process of the proposed method.

**Figure 6:**
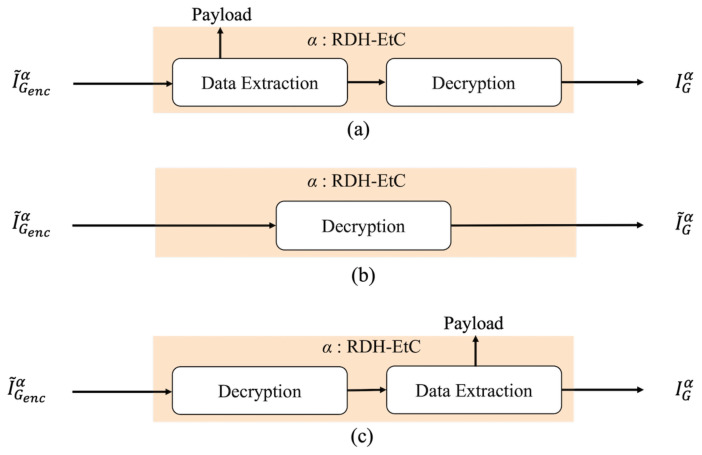
Restoration options for region α. (**a**) Normal, (**b**) decryption without data extraction, (**c**) decryption then data extraction.

**Figure 7:**
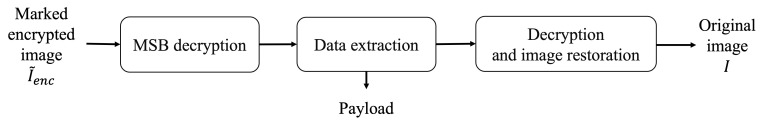
Restoration process of the RDH-MSB method [18].

**Figure 8:**
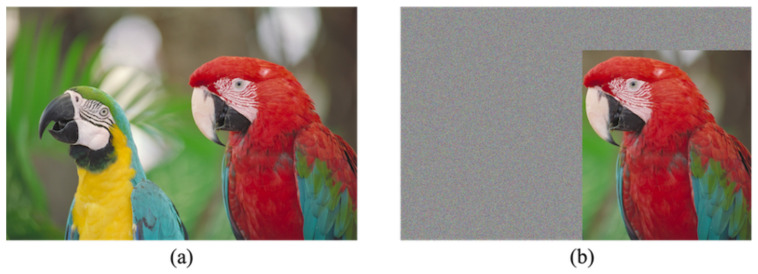
Resulting image with α decryption only (kodim23). (**a**) Original image, (**b**) marked image.

Figure 5 shows the restoration process with decryption and data extraction of the proposed method. Region α has three restoration options, as shown in Figure 6. First, our method can retrieve an original image by conducting the normal restoration process, as shown in Figure 6a. The method has another option for omitting data extraction and only decrypting region α in accordance with Figure 6b. By using this option, we can obtain a marked image containing a payload. In addition, as shown in Figure 6c, region α can be decrypted and the payload then extracted. In the first and third options, an original image is reconstructed. Consequently, in region α, we can obtain a marked image containing a payload after decryption. In contrast, the RDH-MSB method requires extracting a payload in the encrypted domain and thus cannot decrypt marked encrypted images without data extraction, as shown in Figure 7. Figure 8 shows an example of decryption only in region α, which contains the right-side parrot, with the proposed method. It is clear that the decrypted α has high quality despite still containing the payload. Additionally, we can flexibly embed and extract the payload in α; thus, we suppose three types of models. First, a content owner embeds a payload before encryption. In another case, a third party, such as a channel provider, hides a payload, e.g., server information and time stamps, after encryption. In the third model, by dividing α into further fields, the first and second models can be used for each field, respectively. In any case, the embedded payload can be extracted from either plain or encrypted domains.

Here, we consider the security of the proposed method. Note that we suppose the encryption and data hiding algorithms are disclosed to the public. For the case where an attacker desires to obtain visual information on a target image, the robustness against ciphertext-only attacks (COAs), such as brute force attacks and jigsaw puzzle solver attacks, should be discussed. The robustness against COAs for the RDH-EtC method was fully evaluated in [21]. On another front, the RDH-MSB method uses an exclusive-or operation, and thus the robustness depends on key management. Consequently, the proposed method is secure under appropriate key management. In contrast, it is also difficult for the attacker to obtain the content of the payload if every payload is encrypted before data hiding.

## 4. Experimental Results

**Figure 9:**
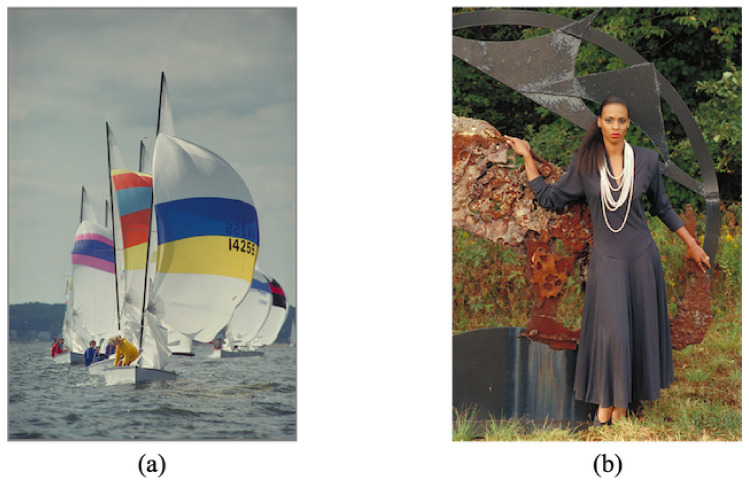
Examples of test images. (**a**) kodim09, (**b**) kodim18.

**Figure 10:**
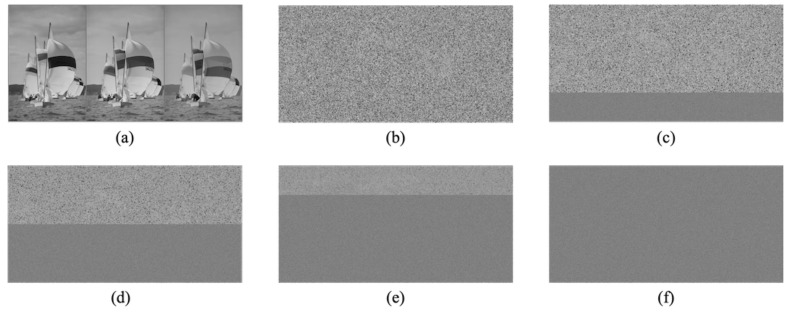
Marked encrypted images with the proposed method (kodim9). (**a**) Grayscale-based image, (**b**) α:β=100:0, (**c**) α:β=75:25, (**d**) α, β=50:50, (**e**) α:β=25:75, (**f**) α:β=0:100.

**Figure 11:**
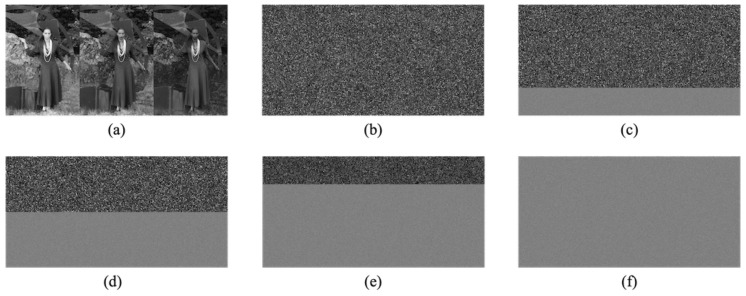
Marked encrypted images with the proposed method (kodim18). (**a**) Grayscale-based image, (**b**) α:β=100:0, (**c**) α:β=75:25, (**d**) α:β=50:50, (**e**) α:β=25:75, (**f**) α:β=0:100.

We confirmed the effectiveness of the proposed method in terms of hiding capacity and lossless compression performance. In the experiments, we used 24 test images from a database of images [28] with 2048×3072 or 3072×2048 pixels. Figure 9 shows two examples of the test images. The original images were split into two regions horizontally; the top and bottom regions were defined as region α and region β in our simulation. To confirm the transition in the compression performance and hiding capacity, we used variable area-ratios for α and β, that is, 100:0, 75:25, 50:50, 25:75, and 0:100; we generated five marked encrypted images for each test image. In practice, a user can classify a target image into two regions more flexibly. We concatenated three color components horizontally in the order of R, G, and B. The block size for encryption in the RDH-EtC method was 16×16 pixels. Figure 10 and Figure 11 show the marked encrypted images obtained by the proposed method.

### 4.1. Lossless Compression Performance and Hiding Capacity

**Figure 12:**
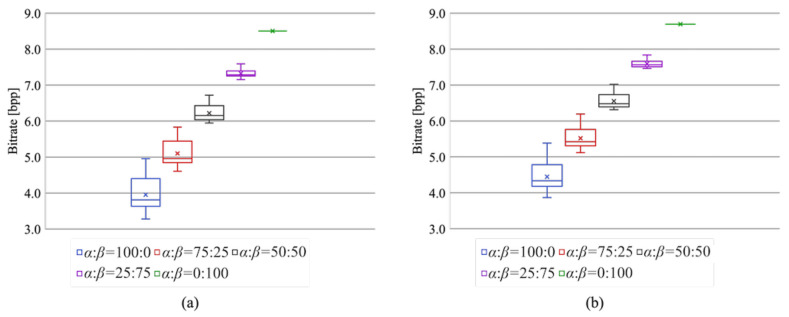
Lossless compression performance. (**a**) JPEG-LS, (**b**) JPEG 2000.

We evaluated the lossless compression performance using JPEG-LS [25] and JPEG 2000 [26]. Figure 12 shows the bitrates of the compressed marked encrypted images. It is clear that the lossless compression performance increased as the area of region α widened. When the area ratio was 0:100, i.e., the RDH-MSB [18] method was used for the entire image, the bitrates with JPEG-LS and JPEG 2000 compression were higher than 8 bpp. This means that the data amount of the compressed images became higher than that of the original images. In contrast, the proposed method can losslessly compress a marked encrypted image by using the RDH-EtC method [21] for a portion of an image. Thus, with the proposed method, the compression performance can be controlled according to the area ratio.

**Figure 13:**
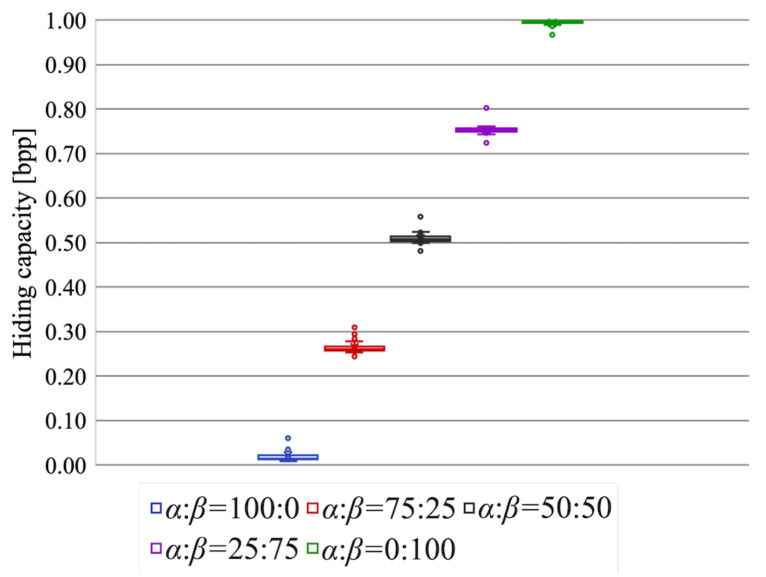
Data hiding capacity.

The data hiding capacity is shown in Figure 13. A higher hiding capacity could be attained as the area of region β became wider. When the area ratio was 100:0, that is, the RDH-EtC method was used for the entire image, the hiding capacity was much lower than 0.1 bpp. Conversely, when the RDH-MSB method was performed on the entire image, the hiding capacity was quite high, close to 1 bpp. The proposed method can enhance the capacity up to around 1 bpp with adjusted area ratios for α and β.

In practice, we have to give information on how to classify an image into two regions to the user. As shown in Figure 3, when region α is chosen with a rectangle shape, the user needs to obtain two coordinates of the top-left and bottom-right pixels of this region. In this simulation, one coordinate is represented by 24 bits, and thus 48 bits are required to locate a single rectangle. If region α contains *n* rectangles, 48×n bits would result in the need for region classification. However, this amount is extremely low compared with the entire hiding capacity. In the case of n=1, 48 bits are approximately 0.001% of the hiding capacity when the RDH-MSB method is applied to the entire image.

In principle, we suppose that region α is chosen with a rectangle shape. When multiple rectangles are concatenated, a different shape can be obtained. If a user expects to use other shapes, it is also easy to introduce triangle and circular shapes. In the case of a circular shape, we store a coordinate of the center pixel and a radius of the circle. When region α consists of rectangle shapes, the data format can be represented as (Number of rectangles = num || ($x_{1}$, $y_{1}$), ($x_{2}$, $y_{2}$), ..., ($x_{num}$), ($y_{num}$)).

**Figure 14:**
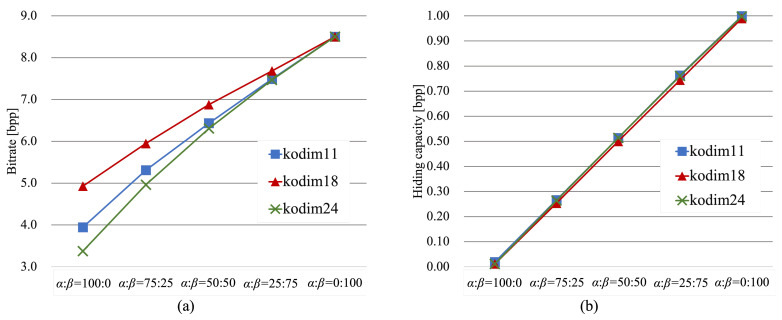
Transition in lossless compression performance and hiding capacity for three images (kodim11, kodim18, kodim24). (**a**) Lossless compression performance by JPEG-LS, (**b**) data hiding capacity.

Figure 14a shows the lossless compression performance using JPEG-LS for three test images, while Figure 14b shows the data hiding capacity for those three images. As shown in Figure 14, the trade-off between the lossless compression performance and hiding capacity shows a linear variation for each image, although each image had a different value and slope in general. As for the lossless compression, the best performance could be obtained when the RDH-EtC method was used for the entire image. In contrast, the highest hiding capacity could be attained when the RDH-MSB method was used for the entire image. The proposed method, however, enables us to combine the RDH-EtC and RDH-MSB methods and thus does not simultaneously accomplish both the highest compression efficiency and hiding capacity. Nevertheless, images can be stored with the minimum amount of data under the required hiding capacity by using the proposed method. The proposed framework could be used in a wider range of fields, including cloud and social networking services.

### 4.2. Comparisons between Proposed Method and Related Work

We show a comparison of the proposed method with the related work in Table 1. The RDH-EtC method can compress marked encrypted images using lossless image coding standards. Further, it can decrypt marked encrypted images without data extraction and thus can derive marked images from them. In addition, it is possible for us to first decrypt marked encrypted images and then extract the payload. The other methods do not have such advantages as the RDH-EtC method. Instead, they have a high hiding capacity, while the RDH-EtC method has a significantly low one. The proposed method utilizes the advantages of both the RDH-EtC and RDH-MSB methods, and it can control the hiding capacity and compression efficiency. Note that since there exists a trade-off between the compression performance and hiding capacity, the proposed method does not simultaneously accomplish both the highest compression efficiency and hiding capacity.

## 5. Conclusions

We proposed a new framework for RDH-EI in this paper. Our method has two main advantages compared with the related work. First, we can store marked encrypted images with the minimum amount of data under the required hiding capacity. An original image is arbitrarily classified into two regions; one region can be highly compressed even after encryption and data hiding, while the other region attains a high hiding capacity. Our method controls the trade-off between the lossless compression efficiency and hiding capacity depending on the user’s request. In addition to the flexible control of the trade-off, we can freely extract the embedded payload before/after decryption in one region. Namely, the decryption process does not require data extraction. Experimental results show that the compression performance and hiding capacity were flexibly controlled depending on the area ratio between two regions. The proposed method, however, is a combination of two RDH-EI methods and thus does not simultaneously accomplish both the highest compression efficiency and hiding capacity.

A constraint on data extraction still remains in the other region, where data extraction must be conducted before decryption. In our future work, we will alleviate this constraint to extend the range of application.

## Figures and Tables

**Table 1:** Comparison of the proposed method with related work.

	Lossless Compression	Decryption without Data Extraction	Decryption Then Data Extraction	High Hiding Capacity
Proposed	🗸	🗸	🗸	🗸
RDH-EtC [21]	🗸	🗸	🗸	×
RDH-MSB [18] Wu et al.’s method [19] Puteaux et al.’s method [20]	×	×	×	🗸

## Data Availability

The data presented in this study are available on request from the corresponding author.
